# Corrigendum

**DOI:** 10.1002/prp2.277

**Published:** 2016-11-24

In Oezel et al. 2016, the correct affiliation for the author Matthias Ocker should be:

Institute for Surgical Research, Philipps University of Marburg, Baldingerstrasse, 35043 Marburg.
